# Assessment of upper limb use in children with typical development and neurodevelopmental disorders by inertial sensors: a systematic review

**DOI:** 10.1186/s12984-018-0447-y

**Published:** 2018-11-06

**Authors:** Irene Braito, Martina Maselli, Giuseppina Sgandurra, Emanuela Inguaggiato, Elena Beani, Francesca Cecchi, Giovanni Cioni, Roslyn Boyd

**Affiliations:** 1 Department of Developmental Neuroscience, IRCCS Fondazione Stella Maris, Viale del Tirreno 331, 56128 Calambrone, Pisa, Italy; 2 The BioRobotics Institute, Polo Sant’Anna Valdera, Viale Rinaldo Piaggio 34, 56026 Pontedera, Pisa, Italy; 3 Department of Clinical and Experimental Medicine, University of Pisa, Via Roma, 56125 Pisa, Italy; 4 Queensland Cerebral Palsy and Rehabilitation Research Centre, Faculty of Medicine, The University of Queensland, Centre for Children’s Health Research, South Brisbane, Australia

**Keywords:** Children, Actigraphs, Inertial sensor, Upper limb, Hand dominance, Asymmetry, Bimanual activity, Typical development, Neurodevelopmental disorders

## Abstract

Understanding development of bimanual upper limb (UL) activities in both typical and atypical conditions in children is important for: i) tailoring rehabilitation programs, ii) monitoring progress, iii) determining outcomes and iv) evaluating effectiveness of treatment/rehabilitation. Recent technological advances, such as wearable sensors, offer possibilities to perform standard medical monitoring. Body-worn motion sensors, mainly accelerometers, have shown very promising results but, so far, these studies have mainly focused on adults. The main aim of this review was to report the evidence of UL activity of both typically developing (TD) children and children with neurodevelopmental disorders (NDDs) that are reliably reported and comparable, using a combination of multiple wearable inertial sensors, both in laboratory and natural settings. Articles were selected from three research databases (PubMed, Web of Science and EBSCO). Included studies reported data on children aged 0–20 years old simultaneously wearing at least two inertial sensors on upper extremities. The collected and reported data were relevant in order to describe the amount of physical activity performed by the two ULs separately. A total of 21 articles were selected: 11 including TD, and 10 regarding NDDs. For each article, a review of both clinical and technical data was performed. We considered inertial sensors used for following aims: (i) to establish activity intensity cut-points; (ii) to investigate validity and reliability of specified markers, placement and/or number of inertial sensors; (iii) to evaluate duration and intensity of natural UL movements, defined motor tasks and tremor; and (iv) to assess efficacy of certain rehabilitation protocols. Our conclusions were that inertial sensors are able to detect differences in use between both hands and that all reviewed studies support use of accelerometers as an objective outcome measure, appropriate in assessing UL activity in young children with NDDs and determining intervention effectiveness. Further research on responsiveness to interventions and consistency with use in real-world settings is needed. This information could be useful in planning UL rehabilitation strategies.

**Electronic supplementary material:**

The online version of this article (10.1186/s12984-018-0447-y) contains supplementary material, which is available to authorized users.

## Background

Upper limbs (ULs) function is fundamental for basic and instrumental daily life activities such as self-care, work, leisure, household routines and social communication. Such activities encompass both gross and fine body movements and involve close cooperation between ULs [1].

The majority of the human manual activities involve two hands playing different roles and working in a coordinated fashion, basilar for the manipulative efficiency [1]. Usually, one hand conducts the action and the contralateral one assists in completing the motor task. As a consequence, the two hands are commonly termed “dominant” and “non-dominant”, respectively [2]. Nevertheless, the left and the right hands are equally important in bimanual activities, since the assisting (or non-dominant) hand defines a steady environment in which the dominant hand can perform a specific activity, such as writing, or actively participates in the movement, like in playing instruments or sports [1], either.

Presence of greater function lateralization to either right or left arm is aimed at optimizing bilateral collaboration between the two sides, referred to as “handedness” [3]. Handedness is complex and influenced by biological, environmental and genetic factors during development. Infants initially use both hands indifferently, but hand preference can emerge very early in development, usually within the first year of age, and becomes progressively more pronounced [4]. This dominance and preference of one of the two hemispheres is directly connected with the body’s preference of handedness and it is immutable [5]. Therefore, handedness can be considered a developmental trait, helping in development of differentiation in motor function and refinement in manual skill, improving the economy and efficiency of the performance and achieving a higher dexterity in handling tools.

Children with NDDs often exhibit a loss of arm-hand function and as a consequence, their handedness is prevented from developing as biologically programmed. Conversely, it results as a trait established “a priori”, depending on the lateralization of the motor disorder. In addition, as one of the upper limbs is impaired, children with NDDs are deprived of the assisting hand contribution, fundamental in the economy of ULs motor performance. Therefore, children show loss of performance, impacting their daily life and resulting in greater dependency, restricted social participation and decreased quality of life [6]. These conditions may be congenital (e.g. unilateral cerebral palsy, UCP) and determine a modification in developmental trajectory or can emerge after a period of well-being (e.g. tremor). In both cases, these impairments dramatically impact daily activity functions, since many of them require the coordinated use of both hands (e.g. dressing, self-care).

Prevalence of handedness can be assessed using self-reporting and surveys (e.g. Edinburgh Handedness Inventory [7] or Waterloo Handedness Questionnaire [8]). However, as hand dominance is a complex phenomenon that relates not only to asymmetrical use, but also to efficiency in terms of speed and accuracy of hand movements, self-reporting often appears to lessen complexity of dominance.

Understanding development of bimanual UL activities in both typical and atypical conditions and in both adults and children is important. Accurate information about UL physical activity could assist clinicians in: i) tailoring rehabilitation programs, ii) monitoring progress, iii) determining outcomes and iv) evaluating effectiveness of treatment/rehabilitation [9]. In this context, recent advances in Information and Communication Technology and related fields (e.g. wearable sensors) offer possibilities in performing standard remote monitoring, that may find many application in various healthcare settings [10]. Body-worn motion sensors such as accelerometers are economical compact and light instruments that allow for an accurate, objective and non-intrusive quantitative measurement of body movements. Previous studies have already reported encouraging results for accelerometers worn on hip and lower limbs to assess physical activity and energy expenditure in both children [11] and adults [12]. Recently, their use has shown good reliability and sensitivity for UL evaluation [13] and has been systematically reviewed for clinical measurement [14], monitoring and provision of feedback in rehabilitation [15]. Results have been very promising but these studies have been mainly focused on adults. However due to ongoing gross motor, manual ability and handedness development, a number of differences exist between child and adult motor abilities. [4].

## Methods

### Research questions

The purpose of this systematic review was to investigate use of a combination of multiple wearable inertial sensors in evaluating UL activity and asymmetry in both TD children and children with NDDs. To pursue this objective, we selected only papers reporting use of at least two inertial sensors worn on both UL, either in laboratory or natural settings.

This systematic review addressed the following issues: 1) How valid are at least two bilaterally worn arm-hand sensors, regarding number and placement of devices, in order to i) describe UL motor capacity and performance in children and ii) investigate UL bilateral asymmetry using accelerometers; 2) Can assessment be generalized across different UL motor abilities and health conditions? 3) What types of accelerometers have been used and how should data be collected and analysed? 4) Do the obtained measurements conform to the outcomes obtained through the administration of standard clinical assessments?

This review has been registered at Prospero CRD42016033687 and follows Preferred Reporting Items for Systematic Reviews (PRISMA) statement for systematic reviews.

### Types of participants

Studies in this review were included if paediatric subjects were evaluated and the established age range included participants from 0 to 20 years, as the US Department of Health and the Food and Drug Administration’ indication [16].

Twofold studies assessing both adults and children were considered, omitting adult sample data and analysis, and focusing on younger participant results only. Studies including both healthy and/or subjects with disabilities were included.

### Outcome measures

Data extraction of outcome measures of interest included amount (raw count) and duration (percentage of recording period) of movements and intensity of physical activity (acceleration vector magnitude, namely 3D summing vector of three axes), performed by ULs to detect potential bilateral asymmetry.

### Criteria for studies inclusion

Searches were conducted between September 2015 and May 2017 (last search: May 16th, 2017) using the following databases: Pubmed, Web of Science and EBSCO (CINHAL®Complete), in English. The following terms were utilized for searches: (actigraph* OR acceleromet* OR inertial) AND (“hand dominance” OR handedness OR “upper limb” OR laterality OR asymmetr* OR “upper extremit*” OR “movement ratio” OR “movement pattern” OR wrist OR hand OR arm) AND (child* OR adolescent OR teen*). Review publications were included. The two first authors (IB, MM) and co-authors (GS, EI, EB, FC) conducted the searches.

### Eligibility

The following criteria were required for inclusion: i) simultaneous data collection of at least 2 inertial sensors, on each UL. Specific placement of inertial sensors could vary across studies (wrist, finger, forearm, etc); ii) report of relationship between recorded data of the two extremities, described as asymmetric coefficient (or similar), comparisons, algorithms, ratios, etc.; iii) participants aged 0–20 years. Exclusion criteria included: i) use of non-human subjects or simulation-based data; ii) use of out-of-centre measurement device to determine physical activity other than inertial sensors (frequency counters, electronic thermometers, Molecular Electronic Transducers (MET), direct observations, questionnaire).

All articles were screened for inclusion by authors (GS, IB, MM, EI, FC), unblinded to manuscript authorship.

### Data extraction

Authors (IB, MM) extracted all data independently (unblinded). Extracted data included both clinical and technical details of included studies.

#### Clinical data

Reported clinical data are as follows: author/journal, year of publication, type of study, potential different measurements used in study other than inertial sensors, number and demographics (age, sex, BMI, height, weight, waist circumference, co-morbid disorders and potential reported details, etc.) of subjects, study setting, inclusion/exclusion criteria of participants, aims, limitations and conclusions. In addition, study quality was assessed (unblinded) by IB and GS using the standards of the QUADAS-2.

#### Technical data

Reported technical data are as follows: make/model and number of inertial sensors, specific placement, wear time, sample acceleration (Hz), data cleaning, threshold to assess intensity of arm movement, threshold as cut-off frequency of filter applied on raw data, main features for accelerometer data comparison, differences between the two hands.

Data for TD and NDD participants were synthesised separately.

## Results

### Study inclusion and assessment

**Fig. 1:**
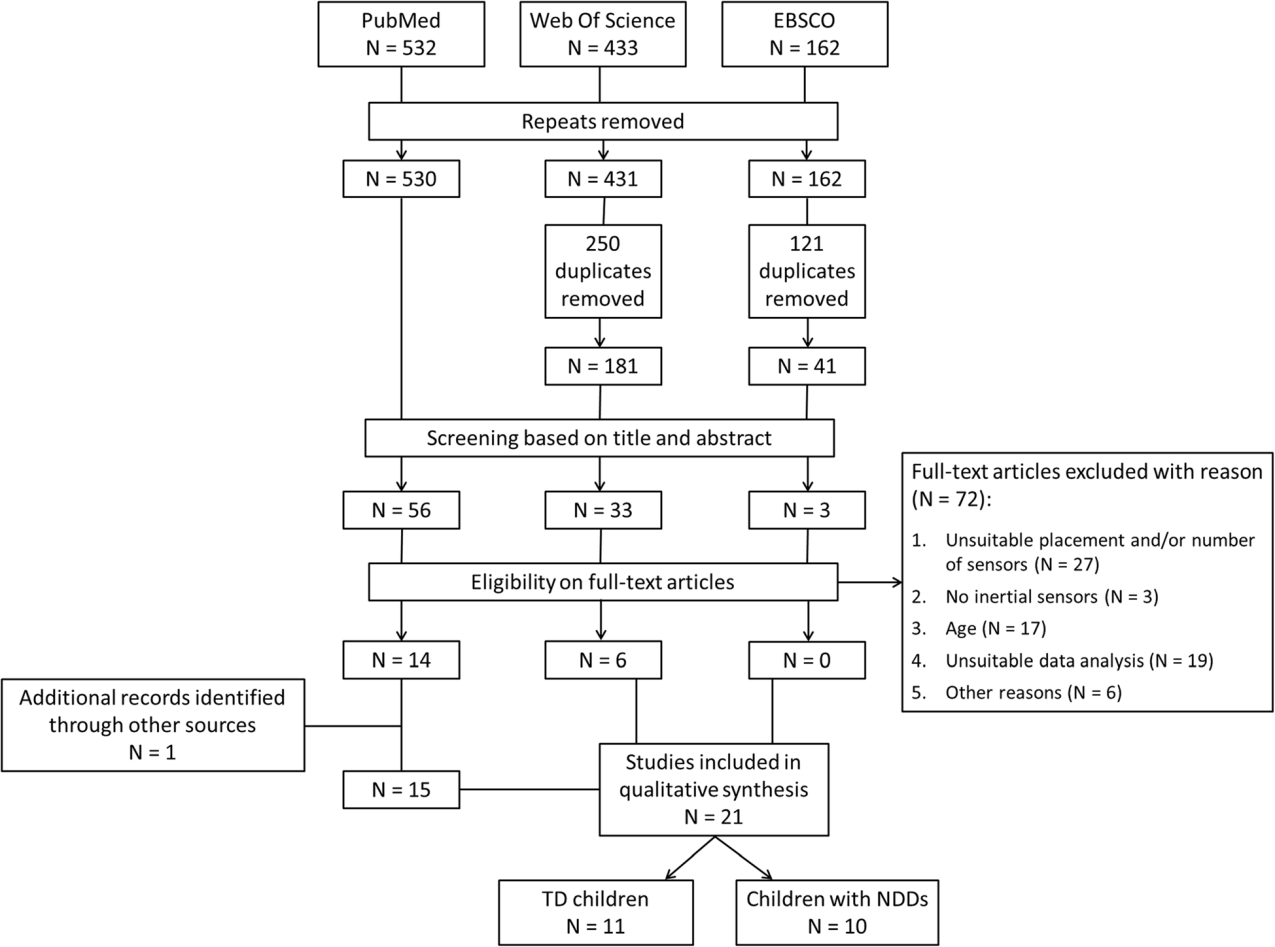
Flowchart of selection of included articles

PRISMA flow diagram is presented in Fig. 1. A total of 1127 articles were identified by initial search criteria. After removing repeats and duplicates, 752 possible records were identified, which were subsequently screened for inclusion/exclusion, according to title and abstract. After evaluation, 92 full articles were retrieved for closer consideration of which 72 did not meet criteria for qualitative review. Reasons for exclusion were grouped into four main categories: 1) unsuitable placement and/or number of sensors: *n* = 27; 2) no inertial sensors (i.e.: inertial eigenvectors, dynamometers, potentiometers): *n* = 3; 3) age (i.e.: adult): *n* = 17; 4) unsuitable data analysis (i.e.: absence of evaluations regarding potential bilateral asymmetry existing between two ULs): *n* = 19; 5) other reasons (i.e.: descriptive texts, unavailability of full article): *n* = 6.

Amongst them, one dissertation was excluded as an unpublished work. However, since it met required criteria of this review, it was later included in analysis, as an additional record, identified through other sources [17].

The remaining 20 articles met full inclusion criteria [18–37]. Number of eligible articles increased exponentially over time. Of these 21 studies, the majority were conducted in the United States (34%), followed by the United Kingdom (19%), Italy, Switzerland and Japan (9% each), and finally France, Israel, Australia and Netherlands (5% each).

### Participant characteristics

Participant characteristics are reported in Tables 1 and 2. Both groups (TD and NDDs) were aged between 2.2 and 20 years. Gender distribution was generally equally balanced. In 11 articles, TD children were enrolled voluntarily and randomly selected from schools, fitness classes and from broad geographical areas [17, 19, 21, 24, 25, 27, 28, 31, 34–36]. The other 10 articles included children with NDDs, whose motor ability, especially ULs, were impaired [18, 20, 22, 23, 26, 29, 30, 32, 33, 37]. Types of NDD are: i) right or left hemiparesis subsequent to cerebral palsy (CP) or postnatal stroke [18, 20, 22, 29, 30, 37], resulting in mild to moderate motor impairment; ii) Attention Deficit Hyperactivity Disorder (ADHD) [32]; iii) Duchenne Muscular Dystrophy [33], including only non-ambulant patients; iv) Niemann-Pick C [23] with moderate ambulatory impairment and moderate to severe disability in one or more functional system (Ataxia, Dystonia, dysmetria, myoclonic jerks, tremor, peripheral neuropathy, etc.); v) paraplegia [26], due to diseases such as myelomeningocele, poliomyelitis, diplegic CP, bone cancer.

**Table 1:** Clinical Data in Typically Developing Children

Author	Study type	Aims	Setting	Sample Size	Mean Age (yrs)	Inclusion Criteria	Exclusion Criteria	Reference Standard
[25] Birmingham A. T. et al. *(1985)*	Survey	To evaluate the variation of tremor frequency and amplitude in relation to the age	Laboratory	109	n = 22 (7–9), *n* = 28 (9–11), *n* = 24 (11–13), *n* = 22 (13–15), *n* = 13 (16–18)	To be attending specific schools or fit classes	NA	NA
[24] Avi Sadeh et al. *(1994) [Study 1]*	Lab-based validation and calibration study	To develop a new sleep-wake scoring algorithm	Laboratory	16	13.8 ± 1.9	Volunteers	NA	NA
[27] Deutsch K. M. et al. *(2006)*	Observational study	To investigate the mechanical and neural components of postural finger tremor	Laboratory	39	*n* = 20 (6.4 ± 3), *n* = 19 (10.5 ± 0.3), *n* = 21 (20.8 ± 1.4)	Volunteers	Neurological disorders, influencing tremor	NA
[34] Graves L.E.S. et al. *(2008)*	Observational study	To examine the contribution of the upper limb and total body movement to adolescents’ energy expenditure whilst playing videogames	Laboratory	13	11–17	Good health picture	NA	NA
[17] Davila E. M. *(2011)*	Observational study	To evaluate the influence of wearing AMs on the D vs ND wrists on measurements of free living PA	Laboratory + outpatient	20	12–17	1) volunteer participants from Bozeman, Montana, 2) 12–17 yrs	NA	NA
[21] Phillips L. R. S. et al. *(2012)*	Lab-based validation and calibration study	To develop physical activity intensity cut-points for use with GENEA accelerometer	Laboratory	44	10.9 ± 1.9	NA	NA	NA
[28] MacArthur B. et al. *(2014)*	2 × 2 mixed design with random allocation.	To measure percentage of time engaged in MVPA and estimated EE with accelerometry in playing AVG vs unstructured OP	ELC playground, ELC room	16 (8 OP vs. 8 AVG)	OP = 6.6 ± 0.7, AVG = 6.3 ± 0.9	1) good health, 2) healthy weight (BMI percentile = 5–85), 3) no limit for physical activity.	Grass allergies. Skin sensitivity to light. Failure to complete all session within a 3-week period.	NA
[19] Lemmens R. J. M. et al. *(2015)*	Cross sectional study	To evaluate the reliability of arm-hand tasks accelerometer records	Laboratory	32	n = 16 children (8.5 ± 1.7), *n* = 16 (14.6 ± 1.5)	Volunteers	Motor problems with arm, hand or shoulder	NA
[31] Kaneko M. et al. *(2015)*	Observational Study	To quantify age-appropriate developmental changes of SNS	Laboratory	233	4–12	Student at the Fukuoka Municipal Elementary School	NA	NA
[35] Dadashi F. et al. *(2015) [Group 2]*	Observational study	To characterise front-crawl swimming skills	Outdoor pool	9	16.0 ± 1.8	Recreational swimmers	NA	NA
[36] Mackintosh K.A. et al. *(2016)*	Observational study	To validate and compare ANNs	Laboratory, semi-structured setting	27	10.8 ± 1.0	Volunteers recruited via a local primary school. The children attended the the laboratory only if: 1) rested state, 2) at least 2 h postprandial, 3) strenous exercise and coffeine avoided in the previous 24 h	NA	NA

*ANNs* Artificial Neural Networks, *AVG* Active Videogames, *EE* Energy Expenditure, *ELC* Early Learning Center, *OP* Outdoor Play, *NA* not available

**Table 2:** Clinical Data in Children with Neurodevelopmental Disorders

Author	Study type	Aims	Setting	Sample Size	Disease	Mean Age (yrs)	Inclusion Criteria	Exclusion Criteria	Reference Standard
[23] Floyd A. G. et al. *(2007)*	Multy-centered study	To analyze the UL motor physiology	Laboratory	15	NP-C	25 ± 10	1) = > 12 years of age, 2) confirmed diagnosis of NP-C by abnormal cholesterol esterification and abnormal filipin staining	1) concurrent enrolment in other clinical trials, 2) drugs or diet supplements, interfering with digestive absorption of study medication, 3) significant history of gastrointestinal disorders, HIV or hepatitis, 4) not comply with study procedures	EDSS
[22] Gordon A. M. et al. *(2007)*	Single-blinded randomized control study	To examine the efficacy of the HABIT	Summer camp	20 (10 HABIT vs 10 CG)	UCP	Total sample = 9.6 ± 6.0, HABIT = 4.5–13.7, CG = 3.9–10.6	1) ability to extend the wrist> 20° and the fingers at the metacarpophalangeal joints> 10° from full flexion, 2) JTHF: > 50% difference between the involved and the non-involved hand, 3) ability to lift the involved arm> 6 in., 4) BBIT = mean score +/− < 1DS	1) health problems unassociated with CP, 2) current/untreated seizures, 3) visual problems interfering with the intervention or testing, 4) MAS > 3.5, 5) orthopaedic surgery on the involved upper extremity, 6) dorsal rhizotomy, 7) botox therapy in the UL in the prior 6 months or within the period of study, 8) intrathecal baclofen.	AHA, BOT-2, CFUS, JTHF
[29] Strohrmann C. et al. *(2013)*	Longitudinal study	i) to monitor children activities in daily life, ii) to evaluate the use of body worn sensors for motor assessment in children	Laboratory	4	CP (2), acquired stroke (2)	10.5 ± 2,12	1) neurological diagnosis leading to stationary stay, 2) age = 5–18 years, 3) cognitive ability to understand the aim of the tasks	NA	Motor Capacity Assessment
[20] Zoccolillo L. et al. *(2015)*	Cross-sectional experimental quantitative study	i) to monitor physical activity during VGT vs conventional therapy, ii) to quantify if VGT enhances number of movements	Outpatient + inpatient	8	UCP	6,6 ± 1,4	1) UCP, 2) 4–14 yrs., 3) GMFCS: I-IV, 4) any Xbox with Kinect at home.	1) IQ < 35, 2) severe comorbidities, 3) incapacity to stand, even with an external support.	QUEST, ABILHAND-kids
[18] Sokal B. et al. *(2015)*	Cross sectional, observational design	i) to evaluate the UL activity, ii) to compare the use of the affected arm between children and adult with hemiplegia	Not reported	28	UCP	3.9 ± 1.7	NA	1) serious or recurring medical complications, 2) spasticity medication within the last 3 months, 3) previous paediatric CIMT, 4) fixed contractures in the affected-arm, 5) invalid accelerometer records (insufficient time, only 1 wrist, unrealistic records, malfunction)	PMAL-R, PAFT
[26] Bergamini E. *(2014)*	Three experimental sessions	i) to identify a biomechanical performance indicators of wheelchair propulsion, ii) develop and assess the efficacy of a specific training program	Basketball court	12 (6 EG vs 6 CG)	Paraplegia (4), myelomeningocele (3), poliomyelitis (2), spastic diplegia (1), below-knee amputation (1), knee arthroprothesis (1)	Total sample = 17.1 ± 2.7, EG = 13–20, CG = 12–20	At least two years of previous wheelchair basketball experience.	Medical contraindications	NA
[32] Kaneko M. et al. *(2016)*	Observational study	i) to establish a quantitative evaluation system of soft neurological signs	Laboratory	33	ADHD	7–11	1) patients of the Kurume University Hospital, 2) positive DSM-IV criteria for ADHD diagnosis, 3) WISC-III > 70	NA	NA
[33] Le Moing A.G. et al. *(2016)*	Observational study	i) to highlight the feasibility of quantifying the range of upper limb movements	Laboratory	7	DMD	18.5+/− 5.5	1) patients of the Institute of Myology, 2) age > 10 years old, 3) non-ambulant, 4) able to sit for at least 3 h in the wheelchair	1) cognitive impairment, 2) occurrence of neurological/inflammatory/infectious/endocrine/acute orthopaedic disease in the precious month, 3) scheduled surgery within 3 weeks of inclusion date, 4) surgery of the upper limbs in the previous three months	MyoSet (MyoPinch, MyoGrip and MyoPlate), BBT, Minnesota Test
[30] O’Neil M.E. et al. *(2016)*	Observational study	i) to evaluate the inter-instrument reliability and concurrent validity of 3 accelerometer-based motion sensors for measuring PA intensity	Clinical standardized setting	57	CP: hemiplegia (29), diplegia (26), quadriplegia (3)	12.5 ± 3.3	1) GMFCS = I-III, 2) ambulatory children, 3) 6–20 years old, 4) able to follow instructions and protocol directions, 5) able to wear 3 pairs of accelerometers and 1 portable indirect calorimeter.	1) recent musculoskeletal injuries, limiting their PA levels, 2) orthopaedic surgery within the precious 6 months, 3) botulinum toxin or phenol injections within the previous three months, 4) previous unstable medical conditions limiting PA levels, 5) unstable emotional or behavioural status.	NA
[37] Cocker-Bolt P. et al. *(2016)*	Prospective pre-test/post-test study	To determine the feasibility and use accelerometers before, during and after a CIMT program	Outpatient + Laboratory	12	UCP	4.9 ± 1.33	1) UCP, 2) able to use the affected UL as a gross assist during play and self-care activities, 3) no significant developmental delays, 4) ambulatory, 5) no additional health impairments	1) significant intellectual disabilities, 2) seizure disorders, 3) botulinum toxin injections in the previous 6 months	MA2

*ADHD* Attention Deficit Hyperactivity Disorder, *AHA* Assisting Hand Assessment, *BOT-2* Bruininks–Oseretsky Test of Motor Proficiency, *CFUS* Caregiver Functional Use Survey, *CG* Control Group, *CIMT* Constraint-Induced Movement Therapy, *DMD* Duchenne Muscular Dystrophia, *EDSS* Extended Disability Status Scale, *EG* Experimental Group, *IQ* Intelligence Quotient, *JTHF* Jebsen–Taylor Test of Hand Function, *MA2* Melbourne Unilateral Upper Limb Assessment – 2, *MAS* Modified Ashworth Score, *NP – C* Nieman Pick C, *PAFT* Pediatric Arm Function Test, *PMAL-R* Pediatric Motor Activity Log – Revised, *QUEST* Quality of Upper Extremity Skills Test, *UL* upper limb, *VGT* Video-Game based Therapy

Examination settings varied across studies as reported in Tables 1 and 2. QUADAS-2 results are reported as Supplementary material (see Additional file 1, Additional file 2: Figure S1, Additional file 3: Figure S2, Additional file 4: Figure S3).

### Forms of assessment

#### Upper limb accelerometer applications

Details about use of inertial sensors in both groups are reported in Tables 3, 4 and Tables 5, 6. Application of accelerometers in selected studies were attributed to several objectives: i) to establish activity intensity cut-points [17, 21, 30, 32]; ii) to investigate validity and reliability of specified models of inertial sensors [19, 21, 23–25, 29, 30]; iii) to examine effect of posture [25], placement and number of sensors [17, 19, 24, 26, 29, 30, 36]; iv) to develop novel monitoring tools, to measure and quantify symptoms, neurodevelopmental delay and/or autonomy of patients suffering from chronic disabilities [18, 20, 22, 23, 26, 29, 32, 33, 37]; v) to evaluate duration and intensity of natural limb movements, defined motor tasks and tremor (in both groups) [17–31, 33–37]; vi) to assess efficacy of pre-specified rehabilitation protocols [20, 22, 28]. Compliance with accelerometers was reported in five studies [17, 26, 30, 33, 36]. They were generally well perceived and tolerated. Only one case [17] reported occasional swelling.

**Table 3:** Technical Data for collection phase in Typically Developing Children

Author	Sensors Number	Sensors Type & Make	Placement	Wear time	Sample frequency
[25] Birmingham A. T. et al. *(1985)*	2	Accelerometers (Bruel and Kjaer type 4367).	Terminal phalanx of each middle finger	3 min for each hand	5-s epoch
[24] Avi Sadeh et al. *(1994) [Study 1]*	2	Actigraphs (AMA-32, Ambulatory Monitoring, Inc., Ardsley, NY).	Each wrist	2 nights (about 7 h to night)	1-min epochs.
[27] Deutsch K. M. et al. *(2006)*	2	Uniaxial wireless accelerometers (Coulbourn T45–10, calibrated on each day of testing).	Dorsal surface of the tip of the distal segment of each index finger.	three 10-s consecutive trials, about 5 s breaks between trials.	200 Hz
[34] Graves L.E.S. et al. *(2008)*	6	1) Actiheart (Cambridge Neurotechnology Cambridge, UK), 2) 4 uniaxial ActiGraph accelerometers (GT1M, Fort Walton Beach, FL, USA)	1) on the skin at the base of the sternum, 2) on the midaxillary line of the right and left hip and on each forearm proximally from the wrist joint .	60 min	2) 30 Hz
[17] Davila E. M. *(2011)*	2	Actical triaxial AMs (Respironics Co., Inc., Bend, OR, USA).	Dorsal side of each wrist	Full seven days (24 h/day).	15-s epoch
[21] Phillips L. R. S. et al. *(2012)*	3 + 1	Triaxial wireless accelerometers GeneActive (Unilever Discover, Colworth, UK) + ActiGraph GT1M (Actigraph, Pensacola, FL, USA).	Each wrist and + right hip (ActiGraph GT1M worn adjacent to the hip mounted GENEA)	Activities: 5 min; Lying supine: 10 min.	GENEA: 80 Hz, ActiGraph GT1M: 1 s epochs.
[28] MacArthur B. et al. *(2014)*	3	Actical accelerometers (Actical, Philips Respironics Co. Inc., Bend, OR).	Each wrist + hip	20 min	15-s epoch
[19] Lemmens R. J. M. et al. *(2015)*	7	Sensor devices, composed by a triaxial accelerometer, triaxial gyroscope, triaxial magnetometer (SHIMMER Research, Dublin, Ireland).	Chest + Dominant and non-dominant arm-hand: on the dorsal side of the hand, of the wrist and on the upper arm	Not specified.	128 Hz
[31] Kaneko M. et al. *(2015)*	4	Wearable sensors composed of three-axis acceleration and three-axis angular velocity sensors (WAA-006, WAA-010, ATR-Promotions, Kyoto, Japan)	Both hands and elbows	Four motor tasks: 10 s for each task	100 Hz
[35] Dadashi F. et al. *(2016) [Group 2]*	3	Waterproof IMUs (Physilog III, BioAGM, CH, 3D accelerometer, 3D gyroscope).	2 IMUs placed on the dorsal side and distal end of the forearms, one on the sacrum.	Not specified.	500 Hz
[36] Mackintosh K.A. et al. *(2016)*	9	Triaxial accelerometer (Actigraph wGT3X+, Florida, USA)	On the lateral plane of each ankle, knee, hip, wrist, and centre of the chest.	30 min	100 Hz

*IMU* Inertial Measurement Unit, *Hz* Herz

**Table 4:** Technical Data for analysis phase in Typically Developing Children

Author	Accelerometer data comparison	Differences between the two hands	Data cleaning	Threshold (cutoff frequency of filter applied on raw data)	Threshold to assess the intensity of arm movement
[25] Birmingham A. T. et al. *(1985)*	RMS of tremor amplitude, dominant peak and its frequency.	For rest tremor, amplitude in the dominant hand was significantly lower in adolescence and early adult life than in childhood, for the non-dominant hand the statistically significant difference was sustained to later life. For work tremor, dominant hand frequency declined significantly with age, both hands continue to decline in adulthood.	Frequency analysis of the tremor waveform was filtered to remove frequencies above 50 Hz to prevent alias contamination	50 Hz	NA
[24] Avi Sadeh et al. *(1994) [Study 1]*	Accelerometric data matched with PSG scoring performed to develop the scoring algorithm: PS probability of sleep	The mean activity level of the dominant wrist was significantly higher than that of the nondominant wrist during PSG-determined sleep (6.84 vs. 6.16), as well as during wakefulness (25.8 vs. 22.3).	NA	NA	NA
[27] Deutsch K. M. et al. *(2006)*	The peak frequency within two frequency bands (5–15 Hz and 15–30 Hz) and the proportion of power exhibited at the peak frequency determined (based on power spectral density calculated using Welch’s averaged periodogram method).	The peak frequency of the finger of the dominant hand (21.4 Hz) was higher than nondominant hand (20.7 Hz) in the 15–30 Hz frequency band. No significant differences in proportion of power exhibited at peak frequency within the 5–15 Hz of postural tremor as a function of age, hand dominance or hand configuration. Postural tremor of nondominant hand was significantly more regular than dominant hand.	Band-pass filtered	1 Hz - 50 Hz	NA
[34] Graves L.E.S. et al. *(2008)*	Means and standard deviations of activity counts (counts/min)	Activity of the dominant limb was significantly greater than non-dominant during tennis and bowling (*P* < 0.001) and non-dominant limb activity was significantly greater during boxing than bowling or tennis (*P* < 0.001). Activity counts from the left wrist for tennis and boxing (*r* = 0.710 and 0.744, *P* < 0.01) and the right wrist for boxing (*r* = 0.586, *P* < 0.05) were significantly correlated with EE.	Band pass filtering	0.21–2.28 Hz	NA
[17] Davila E. M. *(2011)*	Data Trasformation: AEE, Time. Data Summarization Characteristics: Bouts Duration, Intensity Thresholds.	No statistical differences between outcome variables for any bout duration (1, 5, 10 min) within L and MV intensity categories between AMs (D versus ND, LW versus RW) or model (1R versus 2R). Dominant and RW AMs were no-significantly higher than ND and LW, respectively, within MVPA intensity. In contrast, ND and LW AMs were non-significantly higher than D and RW within L intensity PA. Identical results within gender.	Quantity control checks were performed to identify periods on non-wear.	NA	Light (AEE < 0.05 kcals/kg/min), moderate (0.05 < AEE < 0.09 kcals/kg/min), vigorous (AEE ≥ 0.10 kcals/kg/min).
[21] Phillips L. R. S. et al. *(2012)*	VM with gravity-substracted.	Both sides demonstrated good criterion validity (right: *r* = 0.9, left: *r* = 0.91) and good concurrent validity (right: *r* = 0.83, left: *r* = 0.845). ROC analysis proved GENEA monitors able to successfully discriminate among all intensity levels.	NA	NA	Sedentary (< 1.5 METs), light (1.5–2.99 METs), moderate (3–5.99 METs) and vigorous (≥ 6 METs). The accelerometer counts for activities were coded into binary indicator variables (0 or 1) based on intensity.
[28] MacArthur B. et al. *(2014)*	Percentage of time in MVPA calculated by summing the number of 15-s intervals in which the activity counts were ≥ 574 counts/15 s.	The accelerometers placed on the wrists did not find differences in the conditions in percentage MVPA (right: 48.8 ± 29.5%, left: 47.6 ± 28.8%).	NA	NA	MVPA: activity counts ≥574 counts/15 s.
[19] Lemmens R. J. M. et al. *(2015)*	ICC parameter (based on VM).	Within-subject reliability calculated for the 2 arm hands separately, median ICCs ranged between 0.68–0.92. Between subject reliability for the 2 arm hands separately, median ICCs ranged between 0.61–0.90.	Zero time-phase, low-pass filtered	1.28 Hz	NA
[31] Kaneko M. et al. *(2015)*	Postural stability of the hands and elbows, rotational speed, mirror movement, two parameters of bimanual symmetry, compliance	All indices had a tendency to increase with age.	Low-pass filter	6 Hz	NA
[35] Dadashi F. et al. *(2016) [Group 2]*	Average propulsive phases of right and left arms, pull and push phases (Δpull, Δpush), sum of aerial recovery and entry catch phases (ΔNProp), index of coordination (IdC).	By increasing the velocity, the duration of arm under-water phases (Δpull + Δpush) and accordingly IdC did not change significantly. G2 group used 2,8% lower catch-up pattern (P < 0,01) by increasing the arm under-water phases (*P* < 0.016) and using 6.5 more arm stroke (P < 0.001). No changes in the stroke length and cycle velocity variation were observed (*P* > 0.22).	NA	NA	NA
[36] Mackintosh K.A. et al. *(2016)*	Mean and variance of the accelerometer counts in each 15 s window. These extracted features were used as inputs into the ANNs, a specific type of machine learning model. RMSE.	The ANNs for left and right wrist accelerometers had a lower correlations with predicted EE. No significant differences in RMSE analysis. Despite significant advantages in terms of compliance, they could lead to potentially marginal losses in EE prediction accuracy.	NA	NA	1,4% of collected data were removed when EE < 0,5 MET (measured with MetaMax 3B)

*AEE* Activity Energy Expenditure, *AM* Activity Monitors, *ICC* Intraclass Correlation Coefficient, *IMU* Inertial Measurement Unit, *MET* Molecular Electronic Transducers, *PSG* Polysomnography, *RMS* Root Mean Square, *RMSE* Root Mean Square Error, *ROC* Receiver Operating Characteristic, *VM* Vector Magnitudes

**Table 5:** Technical Data for collection phase in Children with Neurodevelopmental Disorders

Author	Sensors Number	Sensors Type & Make	Placement	Wear Time	Sample frequency
[23] Floyd A. G. et al. *(2007)*	2	Piezoresistive uniaxial accelerometers with linear sensitivities of 4.5 mV/g in the biological tremor range (0–25 Hz)	Over the dorsum of both hands	Multiple recording and total recording time lasted 1–2 h	300 Hz
[22] Gordon A. M. et al. *(2007)*	2	Accelerometers (Manufacturing Technology Inc. Fort Walton Beach, FL, model 7164)	Each wrist	During the AHA test session	10 Hz
[29] Strohrmann C. et al. *(2013)*	10	ETH Orientation Sensor (ETHOS) = IMU composed by a 3D accelerometer, a 3D gyroscope and a 3D digital compass. Not commercially available.	Upper (wrists and upper arms) and lower extremities (upper legs and feet) and the trunk.	1 h, once per week over a course of four weeks.	100 Hz
[20] Zoccolillo L. et al. *(2015)*	5	Wireless triaxial accelerometers (Trigno, Delsys®).	Posterior part of forearms, of shanks and of lower trunk in correspondence of the centre of mass (L2-L3).	During 5 continuous minutes of video-game based therapy and 5 min of CT.	Not specified
[18] Sokal B. et al. *(2015)*	2	Biaxial wireless accelerometers (Model 71,256, Actigraph, Pensacola, FL)	Dorsal side of both wrists just above the styloid process	During waking hours for at least 9 h daily for 3 consecutive days after the testing session.	10 Hz, integrated over a user-specified epoch (2 s).
[26] Bergamini E. *(2014)*	3	IMUs (Opal, APDM Inc., Portland, Oregon, USA).	Both wrists and backrest of the wheelchair.	Time was manually recorded. Total time not reported.	128 Hz
[32] Kaneko M. et al. *(2016)*	4	Acceleration and angular velocity sensors (WAA-006, WAA-010, ATR-Promotions, Kyoto, Japan)	Both hands and elbows	Two motor tasks (imitative motor task and a maximal-effort motor task): 10 s for each task	100 Hz
[33] Le Moing A.G. et al. *(2016)*	2	Watch-like devices contained a three-axis accelerometer, a three-axis gyroscope, and a three-axis magnetometer	On each wrist	At least 30 min to complete all the tasks, without concerning potential resting period	NA
[30] O’Neill M.E. et al. *(2016)*	6	1) StepWatch activity monitor (uniaxial), 2) Actigraph GT3X (triaxial), 3) BodyMedia SenseWear Pro Armband (triaxial).	1) superior to the left/right malleolus, 2) on a waist elastic belt superior to the right/left iliac crest, 3) dorsal side of each upper arm at the midbelly of the triceps muscle	During each data collection, lasting 2–2,5 h	1 s for ActiGraph, 3 s for StepWatch, and 60 s for SenseWear.
[37] Coker-Bolt P. et al. *(2017)*	2	Triaxial Actigraph GT9X Link (Actigraph, Pensacola, FL)	On each wrist	6 h a day before and after the CIMT program (tot: 12 h)	30 Hz

**Table 6:** Technical Data for analysis phase in Children with Neurodevelopmental Disorders

Author	Accelerometer data comparison	Differences between the two hands	Data cleaning	Threshold (cutoff frequency of filter applied on raw data)	Threshold to assess the intensity of arm movement
[23] Floyd A. G. et al. *(2007)*	Side-to-side relationship of tremor amplitude, peak tremor frequencies and amplitude variability.	Action tremor amplitudes were relatively symmetric between the dominant and non-dominant hands, postural tremor was not symmetric bilaterally (3 of 8 patients were unilateral), amplitudes of bilateral cases correlated within subjects.	In the FTN trials only, frequencies below 2 Hz were excluded	2 Hz	NA
[22] Gordon A. M. et al. *(2007)*	Percentage of hand use (activity counts)	The percentage of use of involved extremity remain the same in controls, 70% of the task performance, while increased from 62.6 to 77.8% for the children who received HABIT (not correlate with the change in AHA scores). Use of the non-involved extremity remained the same across testing sessions in both groups.	NA	NA	NA
[29] Strohrmann C. et al. *(2013)*	TIME, mean value of MI, MIV, DF, SM, ARE, RANG, ArmSync, gait parameters (all based on VM).	MIV is larger for the unaffected hand, the energy associated to the dominant frequency of the affected hand vs. unaffected hand was much lower, the SM parameter of the unaffected side vs. affected side was twofold.	Low-pass filtered	45 Hz	NA
[20] Zoccolillo L. et al. *(2015)*	RMS of acceleration.	Hemiparetic side was moved less than healthy side. In VGT the paretic side was moved − 20 ± 13% less than the other side, while this difference was not significant in CT (− 10 ± 28%).	Low-pass filtered and after the mean substraction for removing the contribution of gravity acceleration.	20 Hz	NA
[18] Sokal B. et al. *(2015)*	Duration SV, duration ratio SV, intensity SV, intensity ratio SV.	Partecipants moved their more-affected arm for 55.7% and their less-affected arm for 64.9%, ratio 0.86. The intensity of more-affected arm was 41.3 counts/s and for less-affected arm was 60.5, ratio 0.71.	Segments when partecipants appeared to have removed the accelerometers were removed.	NA	Raw values for each 2 s recording epoch were dichotomized around a low threshold (i.e., 2) with above-threshold values set to a positive costant and at- or below-threshold values set to zero.
[26] Bergamini E. *(2014)*	Symmetry index, a peak of the acceleration magnitude and CV (all based on VM).	Symmetry index: - CG: ES2 (48.92%) and ES3 (47.86%), − EG: ES2 (47.77%) and ES3 (48.62%). These values indicate good symmetry.	Low-pass filtered	12 Hz	NA
[32] Kaneko M. et al. *(2016)*	Rotational speed, mirror movement, postural stability of rotating elbow, temporal change of rotational size in each index, bimanual symmetry, compliance.	All scores of ADHD children was lower than TD children. In bimanual symmetry the score of ADHD children increased with age and was significantly different to TD aged 8 and 10 years old. The variability of children’s score in compliance and temporal change of rotational size in ADHD vs. TD was larger.	Low-pass filtered	6 Hz	NA
[33] Le Moing A.G. et al. *(2016)*	Norm of the angular velocity, ratio of the vertical component of the acceleration, model-based computed power, elevation rate	Not find any side effect between the dominant and non-dominant hands. Patients performed better with their dominant side but this was not statistically significant, due to the small size of the population and the advanced stage of the disease.	NA	NA	NA
[30] O’Neill M.E. et al. *(2016)*	Median (IQR) evaluated and compared between right and left side for each parameter and each device, ICC, CIs	Each accelerometer is stable in data collection on both sides, indicating that movement asymmetries may not influence PA measures. Because all 3 accelerometer models exhibited excellent inter-instrument reliability for measuring PA in a variety of real-world activities in TD, it may be appropriate also for CP to wear accelerometers on the right side.	NA	NA	NA
[37] Coker-Bolt P. et al. *(2017)*	Active duration, mean activity count, use ratio and magnitude ratio (all based on VM, down-sampled to 1 Hz).	Significant increase in the duration and mean actvity count of affected upper limb use during each camp day and in three of five days in comparison to pre-test data, respectively. No significant changes in all scores pre- vs. post-CIMT.	NA	NA	Upper limb activity when the vector sum activity count > 0.

*ARE* Average Rotation Energy, *ArmSync* Synchrony of Arm Movement, *CIs* Confidence Intervals, *CT* Conventional Therapy, *CV* Intercycle Variability, *DF* Dominant Frequency, *FTN* finger – to – nose, *IQR* Interquartile Range, *MI* Movement Intensity, *MIV* Movement Intensity Variation, *RANG* Range of Angular Velocity, *SM* Smoothness of Movement, *SV* Summary Variable, *TIME* Task Completion Time

##### Accelerometer type, site and duration

Included studies were grouped by: i) sensor type, ii) placement, depending on whether they were placed only on ULs or elsewhere and iii) wear time. When reported, several different inertial sensors were used: tri-axial (6 studies [17, 20, 21, 30, 36, 37], two-axial (1 study, [18]), and uni-axial accelerometers (3 studies, [23, 27, 34]). Some studies used inertial measurement units (IMUs) (6 studies [19, 26, 31–33, 35]), or ETHOS (1 study, [29]), namely IMU composed by a 3D accelerometer, 3D gyroscope and 3D digital compass.

UL sensor placement was mainly on dorsal side of both wrists [17, 18, 22–24, 33, 37], or terminal phalanx [25, 27] or each elbow [31, 32]. Additional sensors were placed on hip [21, 28], back [35], lower extremities [20] trunk [29, 36] and chest [19] or externally on wheelchair backrest [26].

Wear time varied across studies (Tables 3 and 5) from 30 min to 9 h a day, from 1 to 7 days. Various activities were performed including resting, walking, writing, assessment or intervention.

##### Accelerometer data collection and analyses

Data were collected either as summed acceleration counts over a specified period, or as dichotomized data representing duration of active and inactive periods [18, 37]. Various approaches were reported for defining a unit of arm activity, with data capture epochs varying from one second to one minute. For analyses, five studies use a 3-D resultant vector (usually termed “vector magnitude”) calculated by applying Pythagorean theorem to each time point of x-, y- and z-components of signals of each sensor [19, 21, 26, 29, 37], three studies use tremor amplitude obtained by accelerometer data [23, 25, 27], and another one use root mean square of acceleration computed on signals for each device [20].

In two studies participants were asked to keep a diary to record wear time and modes and activity periods to assist in analysis of accelerometer data [17, 18].

Included studies were grouped by outcome measures for accelerometer data comparison between ULs. Four studies compared UL movement using arm movement intensity. Sokal, B., et al. [18] evaluated asymmetry as ratio of intensity of more-impaired to less-impaired arm movement, whereas arm movement intensity was quantified by dividing sum of raw recordings by sum of threshold-filtered recordings for each arm. Three studies compared arm movement intensity after defining intensity categories from light (L) to moderate-to-vigorous physical activity (Sedentary (< 1.5 METs), light (1.5–2.99 METs), moderate (3–5.99 METs) and vigorous (≥ 6 METs)) [17, 21, 28].

Comparison of UL movement amount was done by root mean square [20], by means and standard deviations of activity counts (counts/min) [34, 36, 37], by activity counts along with a synchronized video to determine percentage of time each hand was used while performing activities [22] or by elaborating an algorithm matching actigraphy data with polysomnography [24].

Other studies evaluated arm movement asymmetry on the basis of features extracted from sensor data that represent bilateral symmetry [26, 29], including additional features extracted from sensors on feet, upper legs and wrists [29]. Other variables that were suitable and consistent for the study of ULs were considered in the study of Duchenne Muscular Dystrophy patients [33]. The following variables were selected: 1) norm of angular velocity of wrist wearing the device (°/sec), 2) ratio of vertical component of acceleration to overall acceleration, 3) a model-based power calculated as scalar product of torque and angular velocity (W/kg), 4) elevation rate corresponding to temporal derivative of elevation angle of forearm, which represents orientation of device (°/sec). Two Kaneko studies [31, 32] calculated two parameters of bimanual symmetry: correlation coefficient of acceleration along Z axis between right hand and left hand, and time delay of acceleration in Z axis between right hand and left hand. In addition to these parameters, these studies quantified other four parameters to evaluate various characteristics of pronation and supination: postural stability of hands and elbows, rotational speed, mirror movement, and compliance. These parameters were calculated on the basis of peak frequency of continuous fast Fourier transform and absolute value of total sum of acceleration along X and/or Z axis.

Finally, reliability of accelerometer data collection was further confirmed using Intraclass Correlation Coefficient (ICC) parameter [38]. ICC described a good/very good within-subject reliability (0.68 < ICCs< 0.92) [19], between-subject reliability (0.61 < ICCs< 0.90) [19], and inter-instrument reliability (0.94 < ICC < 0.99) [30].

Some relevant implications concerning data collection and analysis arose from several articles. First, reliability might be influenced by low-pass filtering that reduced original signal content. Another factor that might influence between-subject reliability was placement of sensors on the body, directly affecting x, y and z vectors of sensor signals. In order to reduce this problem, it would be useful to calculate a 3-D resultant signal. The resultant (directionless) signal would be insensitive to small positioning differences of sensor on a body segment [19]. Another restraint was revealed when many features are used in a regression model, which means that estimation is very good, but generalization is limited when many data are included. Therefore, to avoid overfitting, it would be desirable to include only significant features in the regression model [29]. Moreover, selecting high frequencies of data collection is recommended in order to obtain precise pattern recognition approaches to classify activity type [21]. Finally, sensor variables were well representative of movements performed during tasks [33], although a primary calibration procedure was needed.

#### Type of outcome measures

Studies included both subjective and objective physical activity and energy expenditure measures.

In seven studies, child motor function was extensively and reliably assessed by experts, using direct observations of uni- and bimanual tasks, such as Quality of Upper Extremity Skill Test (QUEST) [20, 39], Assisting Hand Assessment [22, 40], Melbourne Unilateral Upper Limb Assessment-2 (MA2) [37, 41]. Further appropriate outcome measures were Bruininks-Oseretsky Test of Motor Proficiency (BOT-2), Caregiver Functional Use Survey (CFUS) and Jebsen Taylor Test of Hand Function (JTHF) [22, 42, 43], Pediatric Motor Activity Log – Revised (PMAL-R) [18], ABILHAND-Kids [20, 44], Box and Block Test, Minnesota Test, and MyoSet [33]. In other studies, skilled professionals analysed video recordings using Pediatric Arm Function Test (PAFT) [18, 45], and Motor Capacity Assessment [29], followed by an estimated final general score. Motor Capacity Assessment entailed 10 selected predefined motor tasks from established and validated motor assessments, namely Jebsen Taylor Test of Hand Function (JTHF), Graded and Redefined Assessment of Strength, Sensibility and Prehension (GRASSP), Nine-Hole Peg Test (NHPT), and Timed Up and Go. All are standardized tests aimed at assessing function of both ULs separately. For this reason, these tests and their scores could be used as comparison measures to evaluate and interpret results of accelerometer data analyses. These tests are currently used in clinical practice and are therefore characterized by standardized score ranges, which correspond to different motor capabilities and dexterity levels. As a consequence, reported results for each participant can be reliably used for comparisons to describe accelerometer-based activity intensity brackets and motor patterns, recorded by movement sensors, allowing for inferences about reliability.

In one study [23], patients were rated using Extended Disability Status Scale (EDSS). EDSS was designed for multiple sclerosis patients. Even though it has not been validated for Nieman Pick C, it was used because it is suitable for measuring variability and severity of disability in Nieman Pick C. However, this scale was not analysed further, since it does not represent a standardized physical activity assessment in children.

It was shown that motor capacity measure assessed by PAFT was significantly correlated with intensity and duration of the more-affected arm measured in laboratory [18]. PAFT Functional Ability scale is a reliable and valid measure of more-affected arm motor capacity in CP children between 2 and 6 years old. PAFT test-retest reliability correlation coefficient was 0.74 and convergent validity was supported by a strong inverse correlation (*r* = − 0.6, *P* < 0.001) between PAFT scores and grade of impairment [45].

Other interesting relationships were assessed by QUEST and Assisting Hand Assessment. Significant changes in QUEST after videogame based therapy were related to higher quantitative movements during videogame based therapy [20]. Changes after an hand–arm bimanual intensive therapy were detected by accelerometers used during Assisting Hand Assessment assessments [22]. QUEST is an outcome measure that evaluates movement patterns and hand function in CP children, covering four domains of UL movement (UP dissociated movements, grasp function, protective UP extension and weight bearing), administered and evaluated by a trained physical therapist in a play context. Intra- and inter-rater reliability of QUEST and its domains ranged from 0.86 to 0.96, total score internal consistency was high (α = 0.97) [46]. Assisting Hand Assessment is based on observations of actions performed in relevant and motivating activities. It is composed of 22 items, grouped into 6 main functions for both upper extremities, proposed as a semi-structured play session with specific age-adequate toys and play context, and scored from video recordings using a four-point rating scale by skilled therapists (4 = effective, 3 = somewhat effective, 2 = ineffective, 1 = does not do). Inter- and intra-rater reliability and test-retest reliability were high (ICC = 0.98–0.99) [40].

Another study showed an interesting significant correlation between data acquired by IMUs and motor capacity assessment, a rating assessment performed by independent expert raters from video recordings [29]. In addition, accelerometer data showed a highly significant correlation with “MyoSet”, a tool for measuring hand movement using finger and wrist flexors and extensors by using ‘MoviPlate’ assessment, which consists of alternatively hitting two targets of different heights, placed in a sagittal plane for 30 s and Box and Block Test [33]. Finally, data collected by accelerometers before, during and after a 1–2 week Constraint-Induced Movement Therapy were consistent with clinical scores (MA2), in terms of potential improvements or decreases in quality of affected UL movements [37]. MA2 is a criterion-referenced test of unilateral UP function, validated and reliable for evaluating quality of UP movement in children with neurological impairment aged 2.5 [47] to 15 years. Inter- and intra-rater reliability for this assessment was very high for total test scores (ICC = 0.95 and 0.97, respectively) and moderate to high for individual items (ICC = 0.69–0.91). MUUL showed good internal consistency (α = 0.96) [41].

The most relevant limit of standardized tests is that they require one or more trained therapists to administer assessment and score patients. Moreover, some clinical assessments [18, 20] need, after preliminary testing, further scoring sessions, determined independently from videos by either paediatric physical therapists or occupational therapists.

On the other hand, standardized tests are more sensitive in detecting correlations between quantity and quality of movements. In fact, amount of use can be independent from quality of use [22], because of non-functional movements, such as transition from one position to another, or mirror movements [18].

Another limitation of standardized tests is that they entail a range of tasks carried out in specific environments (capacity) with precise assessments which may not directly reflect how a child really uses affected limb during real-world, day-to-day activities (performance) [48]. Therefore it is important to examine aspects of both capacity and performance of arm use in real-life, everyday activities [37], and to increase the number of tasks, to strengthen validation of generalizability of this approach towards daily life [29].

Finally, some of test tasks need to be adapted in order to meet the functional and muscular abilities of patients so that they are able to complete them [33] and thus reduce frustration levels associated with failure to accomplish tasks [22].

### Use of inertial sensors in TD children

From analysis of reviewed articles on TD children, use of inertial sensors have shown to be applicable for a wide range of measurements of both full body movements, such as sleep cycle, physical activity, energy expenditure, and of more specific UL motor patterns during performance of bimanual activities.

In this regard, the most important and common conclusion derived from studies of inertial sensors in TD children was that there were minimal differences for variables between monitors worn on either left or right UL during gross motor activities. For example, there were no differences in sleep-period data from either the dominant or non-dominant wrist during sleep-wake cycle [24]. Measurement of upper body movement on either limb was similar in activities such as active videogame sessions and outdoor play in terms of differentiating moderate to vigorous physical activity [28].

In one study, 44 TD participants performed a series of activities representative of daily life, such as active videogames, lying supine, seated DVD-viewing, walking and running at various speeds [21]. All were symmetrical activities, concerning upper body segment movements and this was reflected in no significant differences between accelerometer output for wrist-worn actigraphs across age groups.

Similar observations can be seen in two studies of wrist-worn sensors in TD children. The first one confirmed minimal variability in data between wrist sensors worn by either side which were not impacted by gender, activity intensity (low and high), and exercise mode (free living condition or pre-specified physical activities) [17]]. This was backed up in the second study, where repetitive tasks analysed through repeated measure analysis of covariance, controlled for differences in condition and condition order, confirmed no variations between sides [28]. It can therefore be concluded that, in TD children, wrist actigraphy provided reliable recording of each wrist and could become a promising tool for measuring bimanual activities and assessing energy expenditure and physical activity [34].

On the contrary, when asymmetric UL bimanual tasks were considered (such as “drinking from a cup”, “eating with knife and fork”, “combing hair” and “opening a zipper”) appropriate differences between the two ULs were found [19] with a greater variability in movement trajectories for complex tasks compared to simple ones. Consequently, accelerometers showed good reliability also in the studies which explored physiological differences between the two ULs. For instance, discrepancies in trajectories and intensity of arms movements were evaluated with inertial sensors in two groups of swimmers in order to define variability patterns of their techniques. Various movement descriptors were established to identify and compare different performance level groups. As a consequence, inertial sensors could be used also as a tool for refining motor skills [35]. Moreover, when features of physiologic movements in different age groups were detected, it was possible to accurately identify age-related changes occurring in physiologic tremor frequency profiles [25, 27] and in soft neurological signs [31]. Soft neurological signs are minor neurological findings, which are likely to appear in motor performance of typically developing young children, disappearing as they mature.

In conclusion, use of wrist inertial sensors could be useful in assessing motor patterns in children, by delivering important information about amount, intensity and executed motor strategy in specific tasks, such as which side of body or limb is more elicited by the activity itself. It can be hypothesized that this approach could be applied in studies of abnormal motor patterns in children with NDDs.

### Use of inertial sensors in children with NDDs

From the analysis of this systematic review, it can be summarized that inertial sensors are able to distinguish different trends between TD children and children with NDDs. This observation can be supported especially by two studies, in which the same evaluation of hand pronation and supination was administered to a group of TD children and a sample of children diagnosed with ADHD. The obtained movement development curves for both groups were compared. Indices such as bimanual symmetry, rotational speed and postural stability of both hands were lower for children with NDDs than for TD group [31, 32].

Similarly, studies of children with NDDs identified greater UL asymmetry, in contrast to typical bimanual cooperation, characterized by performing activities using both ULs in a balanced fashion. Studies concerning children with UCP assessed use of accelerometry both in uni- and bimanual tasks to evaluate asymmetry between two limbs [18, 20, 22, 29, 37]. Significant differences between impaired and unimpaired UL accelerometer data arose in all articles. It is often observed that the natural tendency would be to compensate with a greater use of non-involved extremity, the greater the severity of impairment. Analysis of collected accelerometer data matched this asymmetric trend and therefore validity was confirmed.

In one study, in order to measure arm synchrony, wheel chair manoeuvring was added, in addition to walking and stair climbing. A smoother pattern was detected by accelerometry in the unimpaired hand, compared to the impaired one, meaning that a performance characterized by several different frequencies indicates a more uncontrolled movement [29]. Changes in clinical outcome measures such as QUEST, Assisting Hand Assessment, MA2 and PAFT after videogame based therapy, HABIT and Constraint-Induced Movement Therapy were related to higher activity intensities and frequency of use recorded by actigraphs [18, 20, 22, 37].

It can therefore be suggested that accelerometer data analysis is able to measure and describe differences in severity of a wide range of pathological conditions. For instance, in movement disorders such as tremor, dystonia, chorea and myoclonus secondary to neurodegenerative disorders (e.g. Nieman Pick C) accelerometry is able to quantify the rate with data related to EMG data [23]. These data may provide important methods in determining severity and progression of pathology and outcome of treatments [23].

Findings of these studies also suggested that the necessary quantity and quality of movement may be related to task complexity, since the laboratory approach did not essentially include tasks performed in a home environment [29]. This point is further strengthened in one study in which the main detected limitation was the clinical setting, even if the protocol was designed to resemble real-world activities as much as possible [30].

In addition, accelerometer data analysis can provide interesting insight into the study of technique patterns and overall test performance, as shown for TD children [35]. For instance, indices such as bilateral symmetry index can help in the study of biomechanical characterization of wheelchair propulsion, identifying potential strengths and weaknesses [26].

A controversial aspect is the relationship between accelerometer data and daily use of ULs in hemiplegia. Several studies in adults with hemiplegia due to chronic stroke have reported strong correlations between amount of movement and use of more-affected arm, while one study in children [18] found no correlation between amount of movement and amount of use of more-affected arm in daily life, suggesting that children differ from adults in this respect. Further studies are needed to explore this topic, and in particular observational studies comparing use of both hands in hemiplegic children with those of TD children. An important aspect could be the presence of mirror movements in hemiplegic children that can reduce differences in the amount of use between the more-affected hand and less-affected one. Another important aspect that should be investigated further is the use of accelerometers to monitor and detect changes during and after UL experimental training. Results in this field are very promising [22, 37] but some are controversial suggesting that changes detected by actigraphs may not be related to those detected by clinical scale [22].

## Conclusions and future perspectives

Understanding development of bimanual UL activity both in TD children and children with NDDs is important, however such knowledge requires further development of quantitative tools such as wearable sensors. This systematic review summarizes the growing body of literature concerning available clinical applications of inertial sensors worn on both ULs in TD children and children with NDDs.

From the analysis of reviewed articles, it can be assessed that, in both groups, inertial sensors are able to detect differences in amount of use between both hands. All reviewed studies support use of accelerometers as a potential future outcome measure, appropriate to measure UL activity in young children with NDDs, to determine intervention effectiveness due to its high interrater reliability and strong concurrent strength with validated capacity measures.

Amongst the different inertial sensors, the triaxial accelerometer is the most commonly used in the articles, and it seems to be the most suitable and reliable to monitor and to collect consistent data about body movement. In addition, collected data are processed to estimate different parameters used to describe various movement features. The majority of them are calculated on the basis of the vector magnitude.

It can be seen that wrist actigraphy is used for several different aims in order to evaluate physical activity of young people. Worn on both ULs, they provide an accurate measure of arm-hand motor patterns and performance regarding unique non-ambulatory activities, such as playing videogames or rehabilitation training. Moreover, when used in a multi-site activity monitoring setup, they are capable of delivering valuable information about relevant features of motor patterns, also regarding analysis of full-body movements. In particular, a minimal setup of three sensors (generally worn on both wrists and hip) are sufficient to cover motor function assessment, in terms of physical activity and energy expenditure. This approach is recommendable also to maximize unobtrusiveness.

Use of inertial sensors exhibits several positive aspects if compared to traditional clinical assessments. Firstly, clinical assessments need to be administered by trained therapists and therefore outcomes can be easily influenced by level of training and experience. Moreover, subjects have to visit the clinic every time they want to check progress, which not only makes the whole process very time consuming but also raises the burden on healthcare costs. Consequently, wrist actigraphy could be introduced as a more affordable and accessible follow-up strategy for a wide number of distant healthcare centres.

A second important feature of inertial sensors is that they can potentially evaluate a comprehensive range of daily activities performed in a home environment. On the contrary, clinical assessments are usually performed in a standardized environment, evaluating a limited number of pre-determined activities. The amount of use and quality of performance of arm-hand related skills measured under laboratory conditions may differ from those performed at home during routine activities.

A further interesting application of inertial sensors is related to extended periods of data collection, considering that they can be worn for long periods of time thus allowing for continuous monitoring.

Common limitations were small sample sizes, lack of control groups, variety of actigraphs and parameters used, so that it was not possible to draw any study conclusions. However, evaluation of differences between dominant and non-dominant UL measured by inertial sensors could play an important role as criteria for evaluating age-appropriate development in neurological functions both in TD children and children with NDDs. Therefore, the accelerometer could be introduced as a reliable assessment tool and as a quantitative evaluation method for developmental disorders.

Further research on responsiveness to interventions and consistency of use in real-life setting is needed. Moreover, additional steps are necessary for outcome measure qualification including demonstration of reliability on day-to-day and week-to-week basis.

This information could be very useful for planning UL activity strategies in interventions.

## Additional files

Additional file 1: QUADAS-2 Results. (DOCX 20 kb)

Additional file 2: **Figure S1.** Risk of bias and applicability concerns summary. The review authors’ judgements about each domain are shown for each included study. (TIFF 239 kb)

Additional file 3: **Figure S2.** Risk of bias graph. The review authors’ judgements about each domain are presented as percentages of the included studies. (TIFF 54 kb)

Additional file 4: **Figure S3.** Applicability concerns graph. The review authors’ judgements about each domain are presented as percentages of the included studies. (TIFF 47 kb)

## Abbreviations

ADHD: Attention deficit hyperactivity disorder
BOT: Bruininks-oseretsky test of motor proficiency
CFUS: Caregiver functional use survey
CP: Cerebral palsy
GRASSP: Graded and redefined assessment of strength, sensibility and prehension
ICC: Intraclass correlation coefficient
IMU: Inertial measurement unit
JTHF: Jebsen Taylor test of hand function
MA: Melbourne unilateral upper limb assessment
MET: Molecular electronic transducers
NDDs: Neurodevelopmental disorders
NHPT: Nine-hole peg test
PAFT: Pediatric arm function test
PMAL-R: Pediatric motor activity log – revised
PRISMA: Preferred reporting items for systematic reviews
QUEST: Quality of upper extremity skill test
TD: Typically developed
UCP: Unilateral cerebral palsy
UL: Upper limb
