# Supplementary material for: Assessment of upper limb use in children with typical development and neurodevelopmental disorders by inertial sensors: a systematic review
Source: J Neuroeng Rehabil. 2018 Nov 6;15:94. doi: 10.1186/s12984-018-0447-y (PMC6219116; doi:10.1186/s12984-018-0447-y)
Supplement: Supplementary file 1 — QUADAS-2 Results. (DOCX 20 kb) [file 12984_2018_447_MOESM1_ESM.docx]

**Supplementary material.**

**QUADAS-2 results**

Concerning the Risk of Bias Section, the most of the included articles fitted our review question, regarding Patient Selection and Index Test Domains (100% and 67% of selected papers, respectively). The “Index test” investigates about the used primary outcome measure and its application in every included study, in order to answer the main questions of the review. In our case it was represented by the usage of at least two movement sensors, attached on both upper limbs, to evaluate the motor asymmetries between each other. In two studies [21; 28] the primary aim was not to compare the data, to draw conclusions about the asymmetry between the two upper arms. Therefore, the Index Test domain of QUADAS-2 was evaluated as at high risk of bias and with high concern of applicability. Nevertheless, the data demonstrated both separately and compared with each other, reason why these papers were suitable to be included in our study. As far as the Reference Standard, both for Risk of Bias and Concerns of Applicability Domains, and, consequently, Flow and Timing Domains, only the 25% of selected papers were suitable. The lack of suitability was mainly related to two main reasons:

1. lacking devices and instruments, developed to identify the differential use of the two upper extremities [17; 19; 25; 28; 32; 36];
2. absence of a suitable reference standard, often due to the different purposes of many of the included articles, compared to our aims [21; 24; 26; 28; 30; 32; 34; 36].

In those studies, which concerned different index tests at the same time, such as accelerometry and sEMG, the QUADAS-2 had been assessed for each index test, but only the suitable index test was considered, to be included for results in this review [23].

Another reason why the Risk of Bias of the Index Test could not be evaluated “Low” was represented by the clearness about the application of a threshold. It was notable that details regarding the threshold used were not clearly specified in five of the manuscripts [22, 24, 30, 33, 35], so that the QUADAS-2 judgement for the Index Test domain in these cases was classified as “Unclear”.

**Supplementary Figure 1.** Risk of bias and applicability concerns summary. The review authors’ judgements about each domain are shown for each included study.

**Supplementary Figure 2.** Risk of bias graph. The review authors’ judgements about each domain are presented as percentages of the included studies.

**Supplementary Figure 3.** Applicability concerns graph. The review authors’ judgements about each domain are presented as percentages of the included studies.
