# Supplementary figures and images for: Assessment of upper limb use in children with typical development and neurodevelopmental disorders by inertial sensors: a systematic review
Source: J Neuroeng Rehabil. 2018 Nov 6;15:94. doi: 10.1186/s12984-018-0447-y (PMC6219116; doi:10.1186/s12984-018-0447-y)

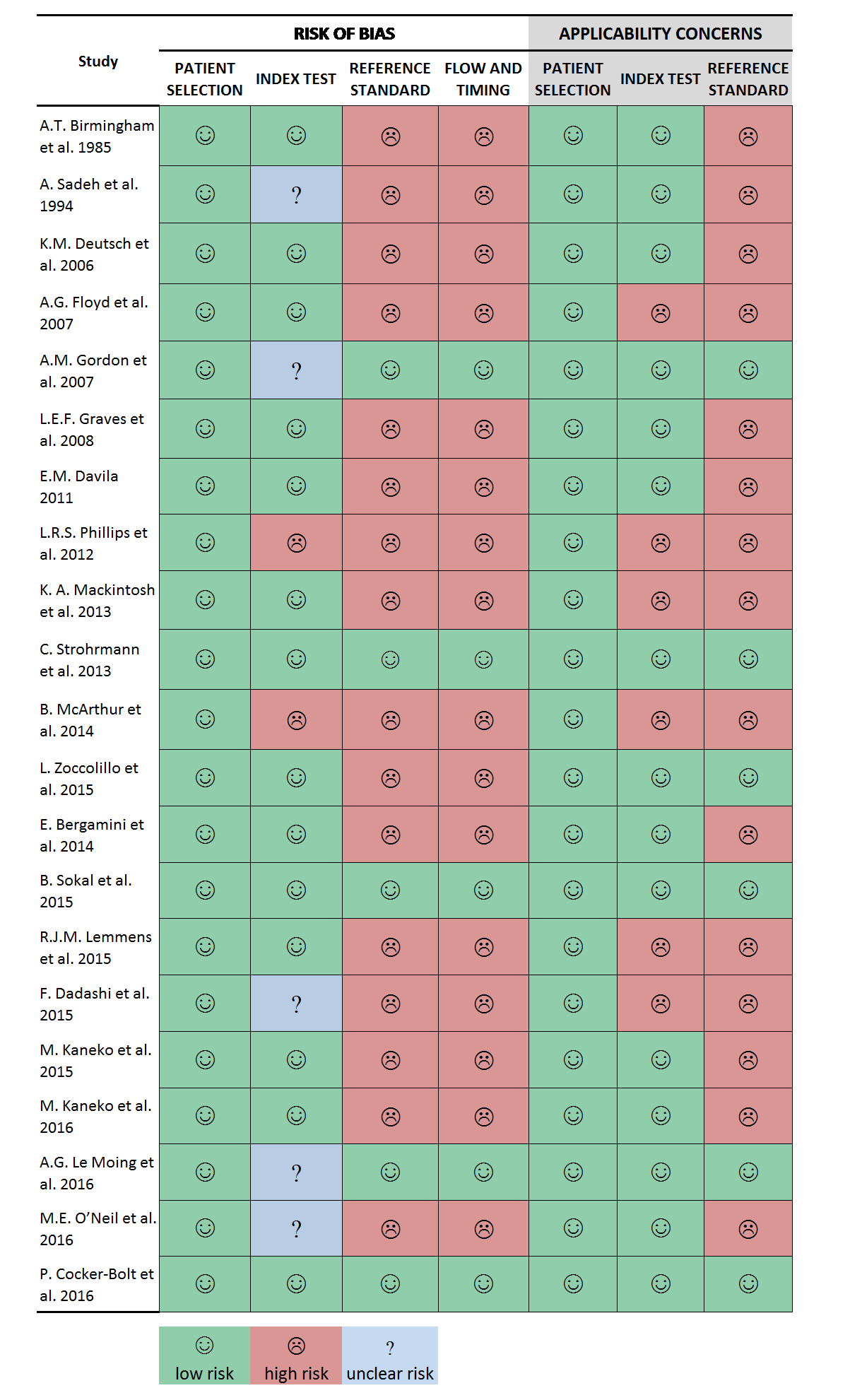

Supplement: Supplementary file 2 — Figure S1. Risk of bias and applicability concerns summary. The review authors’ judgements about each domain are shown for each included study. (TIFF 239 kb) [file 12984_2018_447_MOESM2_ESM.tiff]

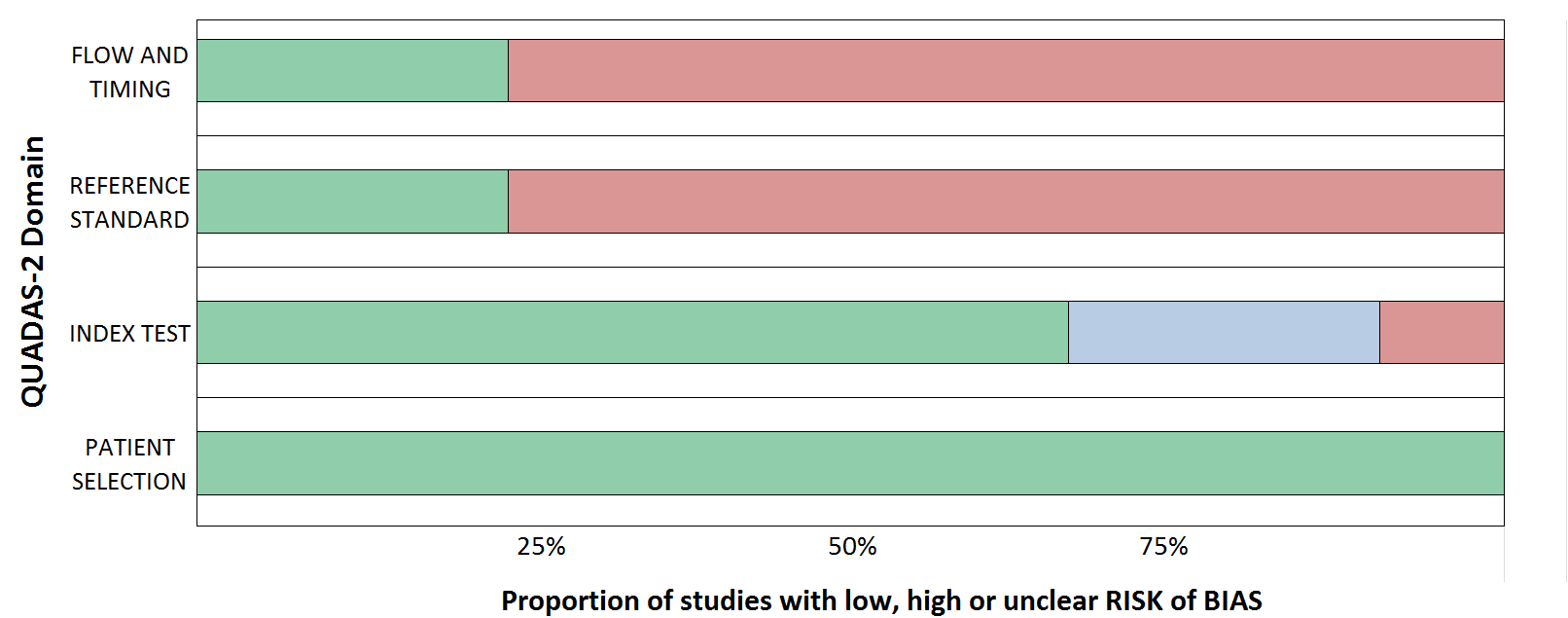

Supplement: Supplementary file 3 — Figure S2. Risk of bias graph. The review authors’ judgements about each domain are presented as percentages of the included studies. (TIFF 54 kb) [file 12984_2018_447_MOESM3_ESM.tiff]

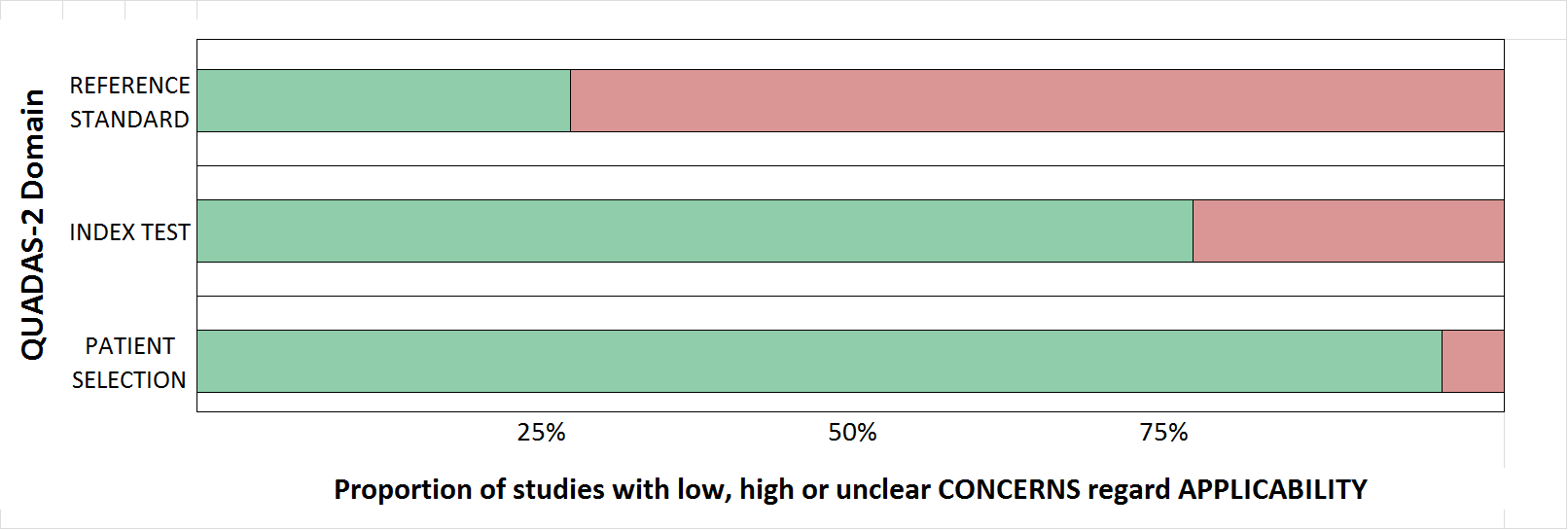

Supplement: Supplementary file 4 — Figure S3. Applicability concerns graph. The review authors’ judgements about each domain are presented as percentages of the included studies. (TIFF 47 kb) [file 12984_2018_447_MOESM4_ESM.tiff]
